# “Patients are not the same, so we cannot treat them the same” – A qualitative content analysis of provider, patient and implementer perspectives on differentiated service delivery models for HIV treatment in South Africa

**DOI:** 10.1002/jia2.25544

**Published:** 2020-06-25

**Authors:** Sophie J S Pascoe, Nancy A Scott, Rachel M Fong, Joshua Murphy, Amy N Huber, Aneesa Moolla, Mokgadi Phokojoe, Marelize Gorgens, Sydney Rosen, David Wilson, Yogan Pillay, Matthew P Fox, Nicole Fraser‐Hurt

**Affiliations:** ^1^ Health Economics and Epidemiology Research Office Department of Internal Medicine School of Clinical Medicine Faculty of Health Sciences University of the Witwatersrand Johannesburg South Africa; ^2^ Department of Global Health Boston University School of Public Health Boston MA USA; ^3^ Department of Epidemiology Boston University School of Public Health Boston MA USA; ^4^ National Department of Health Pretoria South Africa; ^5^ The World Bank Group Washington DC USA

**Keywords:** antiretroviral therapy, adherence guidelines, differentiated Care, HIV, counselling, repeat prescription collection strategies

## Abstract

**Introduction:**

In 2014, the South African government adopted a differentiated service delivery (DSD) model in its “National Adherence Guidelines for Chronic Diseases (HIV, TB and NCDs)” (AGL) to strengthen the HIV care cascade. We describe the barriers and facilitators of the AGL implementation as experienced by various stakeholders in eight intervention and control sites across four districts.

**Methods:**

Embedded within a cluster‐randomized evaluation of the AGL, we conducted 48 in‐depth interviews (IDIs) with healthcare providers, 16 IDIs with Department of Health and implementing partners and 24 focus group discussions (FGDs) with three HIV patient groups: new, stable and those not stable on treatment or not adhering to care. IDIs were conducted from August 2016 to August 2017; FGDs were conducted in January to February 2017. Content analysis was guided by the Consolidated Framework for Implementation Research. Findings were triangulated among respondent types to elicit barriers and facilitators to implementation.

**Results:**

New HIV patients found counselling helpful but intervention respondents reported sub‐optimal counselling and privacy concerns as barriers to initiation. Providers felt insufficiently trained for this intervention and were confused by the simultaneous rollout of the Universal Test and Treat strategy. For stable patients, repeat prescription collection strategies (RPCS) were generally well received. Patients and providers concurred that RPCS reduced congestion and waiting times at clinics. There was confusion though, among providers and implementers, around implementation of RPCS interventions. For patients not stable on treatment, enhanced counselling and tracing patients lost‐to‐follow‐up were perceived as beneficial to adherence behaviours but faced logistical challenges. All providers faced difficulties accessing data and identifying patients in need of tracing. Congestion at clinics and staff attitude were perceived as barriers preventing patients returning to care.

**Conclusions:**

Implementation of DSD models at scale is complex but this evaluation identified several positive aspects of AGL implementation. The positive perception of RPCS interventions and challenges managing patients not stable on treatment aligned with results from the larger evaluation. While some implementation challenges may resolve with experience, ensuring providers and implementers have the necessary training, tools and resources to operationalize AGL effectively is critical to the overall success of South Africa’s HIV control strategy.

## 1 Introduction

South Africa has the largest HIV treatment programme in the world, with 4.7 million public sector patients on treatment and a target of 6.1 million patients by end December 2020 [1]. Data indicate that retention in care and adherence to ART in South Africa is poor [2, 3, 4], with just over 70% of patients starting ART retained in care 12 months later [5]. Rates of viral load suppression are also below the targeted 90% of those on treatment across many districts and facilities [6, 7]. Strategies for early initiation of ART and innovative approaches to treatment adherence are critical as the country’s HIV treatment programme continues to expand [8].

To achieve UNAIDS 90‐90‐90 global HIV targets, countries across sub‐Saharan Africa are developing and scaling up differentiated service delivery (DSD) models for providing antiretroviral treatment (ART) [9, 10, 11, 12]. These targeted, patient‐centred service delivery approaches allow services to be adapted to different patient groups to enable better access to, and outcomes of treatment services, and to increase clinic capacity. In 2015, the South African National Department of Health (NDOH) began implementing the “National Adherence Guidelines for Chronic Diseases (HIV, TB and NCDs)” (AGL) which outline the provision of a minimum package of eight interventions aimed at improving health outcomes along the cascade of care, including linkage to and retention in care and adherence to treatment [13]. In partnership with the NDOH, we conducted an evaluation of five AGL interventions at 12 “early learning” sites to determine whether the AGL interventions were effective in achieving expected outcomes, and to understand the implementation process. The evaluation found that the interventions targeted at stable patients were successful in maintaining suppression and improving retention outcomes among patients [14]. Interventions targeting newly initiated patients or patients not stable on treatment, however, showed little or no improvement in outcomes compared to the standard of care [14, 15]. These findings indicate a need for gaining a better understanding of the AGL implementation through qualitative research. This paper does not present outcomes, rather qualitatively describes the strengths and challenges of the AGL implementation as experienced by health providers, patients and implementing partners in four intervention and four control sites.

## 2 Methods

### 2.1 Description of the intervention

The AGL, developed in 2014, was piloted in 12 early learning sites starting in 2015 in four districts by the government and implementing partners, in order to further inform the national AGL scale up [13]. Provincial governments and implementing partners, typically international or local non‐governmental organizations (NGOs), collaborated to operationalize this guideline across provinces and districts [16]. The AGL interventions target four groups of patients with HIV, those who: (1) are newly testing and initiating treatment (new patients); (2) have initiated treatment and achieved viral load suppression (stable patients); (3) have initiated treatment and have elevated viral loads or missed their schedule for clinical care (patients not stable on treatment); and (4) children and adolescents living with HIV (Table 1) as well as one intervention that focused on facility infrastructure and the provision of integrated care. This evaluation includes only those interventions focused on adults (given the limited scope, of the evaluation we did not include children and adolescents) and excludes the facility infrastructure intervention. The primary intervention targeting new patients is fast track treatment initiation counselling (FTIC). Repeat prescription collection strategies (RPCS), which includes Adherence Clubs (both in and outside of facilities) and decentralized medication delivery (DMD) where medications are delivered to and collected from external pick up points, target stable patients with suppressed viral loads. Enhanced adherence counselling (EAC) and early tracing interventions (TRIC) target those patients not stable on treatment with unsuppressed viral loads or who have missed visits.

**Table 1:** South Africa’s National Adherence Guidelines for Chronic Diseases: approach, interventions and standard of care for each patient groups

Target patient type	Adherence guideline (AGL) intervention [13]	Description of AGL intervention in study districts	Standard of care intervention	Description of standard of care in study districts
**New patients:** Newly testing and initiating treatment	Fast track treatment initiation counselling (FTIC)	Standardized education, counselling, and adherence support for newly diagnosed patients without delaying treatment initiation, and patient assistance to develop their own adherence plan. Patients initiate treatment after the first counselling session and complete remaining sessions at the first and second refill	2015 ART guidelines (NDOH) [13]; Universal Test and Treat (UTT) (from Sept 2016) [17]	Fast‐track initiation for certain patients based on health conditions including pregnant or breastfeeding women, or patients with CD4 ≤ 200 or stage 4 HIV; Start ART as soon patient is ready and within two weeks of CD4 count, regardless of CD4 result or HIV stage
**Patients stable on treatment:** Have initiated treatment and achieved viral load suppression (2 consecutive viral loads <400 copies/mL)	Repeat prescription collection strategies (RPCS)	Clinic‐based Adherence Clubs (AC): Patients receive medication and care during small group meetings (<30) conducted at the clinic or in the community every two months	Community‐based Adherence Clubs (AC) (Implemented as early as 2012) [21]	Implemented in some parts of the country via non‐governmental organizations. Patients receive medication and care during bi‐monthly small group meetings (<30) conducted at a clinic or in the community
		Decentralized medication delivery (DMD): Medications picked up at an external pick‐up‐point every two months	Decanting strategy (from May to June 2016) [14]	Promoted DMD and ACs in effort to decongest clinics. Included only the RPCS from the AGL; no formal launch. Rolled out at other non‐study sites within the study districts
		Spaced fast lane appointment systems (SFLA): Medication collected every two months from a dedicated fast‐lane pick‐up point at the facility with appointment for pick‐up date	2015 ART guidelines (NDOH) [13]	Support groups, SMS reminders, peer support worker, buddy system, community outreach with medication pick‐up at the facility
**Patients not stable on treatment:** Have initiated treatment and have an elevated viral load or missed scheduled clinic visit	Enhanced adherence counselling (EAC)	Enhanced adherence monitoring and targeted counselling interventions for those patients not stable on treatment (as defined by an elevated viral load) with timeous referral for support	2015 ART guidelines (NDOH) [13]	Increased counselling efforts, home visits, social support emphasis (buddy and support group), pill counts
	Early tracing of all missed appointments (TRIC)	Telephonic tracing and home visits by community health workers to trace patients who have failed to return to the facility for scheduled appointments by five days or more	2015 ART guidelines (NDOH) [13]	Telephonic tracing and home visits by CHWs to trace patients who have failed to return to the facility for a scheduled visit by 14 days or more

The AGL guidelines include interventions focused on children and adolescents living with HIV and an integrated care model of patients with chronic conditions.

NDOH staff at district and provincial level, supervised implementation supported by district implementing partners. Each district had at least two partner NGOs supporting AGL implementation [19]. Study staff were not involved in implementation.

### 2.2 Study setting and design

A cluster‐randomized evaluation of the AGL interventions was conducted in 12 health facilities implementing the minimum package of interventions (intervention sites) and in 12 health facilities with delayed AGL implementation (control sites – where implementation of the minimum package of interventions was delayed until scaled‐up in the national rollout) [20]. These sites are located in one district in each of four provinces, representing a range of district profiles in terms of rurality, levels of employment, HIV testing coverage and adherence monitoring (Table S1). Methods and results of the trial are described elsewhere [14, 20].

Nested within the cluster‐randomized evaluation, we conducted a hybrid inductive‐deductive qualitative sub‐study among a sample of patients, providers and implementing partners at one intervention and one control site in each district. The sites were purposively selected from the 24 matched intervention and control sites included in the larger evaluation (the matching of sites is described elsewhere) [20]. In each district the intervention site that first implemented the AGL interventions was selected for this qualitative study along with its matched control site. Semi‐structured in‐depth interviews (IDIs) with health providers and implementers and Focus Group Discussions (FGDs) with patients were done to better understand facilitators and challenges of implementing AGL interventions [21]. We report findings of this study using the Standards for Reporting Qualitative Research reporting guidelines [22].

### 2.3 Analytic framework

Implementation science approaches are critical to understand the mechanisms that underlie the effectiveness of complex programmes [23, 24]. We used salient constructs from the Consolidated Framework for Implementation Research (CFIR) to guide the evaluation, including the design of the data collection instruments and analysis. CFIR categorizes 39 implementation constructs under the main domains of: (1) intervention characteristics (quality, adaptability, complexity); (2) outer setting (patient needs, setting, policies); (3) inner setting (organizational priority, implementation climate, leadership engagement); (4) individual provider characteristics (knowledge about interventions, self‐efficacy, identification with organization) and; (5) process (planning, engaging, executing and evaluating) [25, 26, 27].

**Figure 1:**
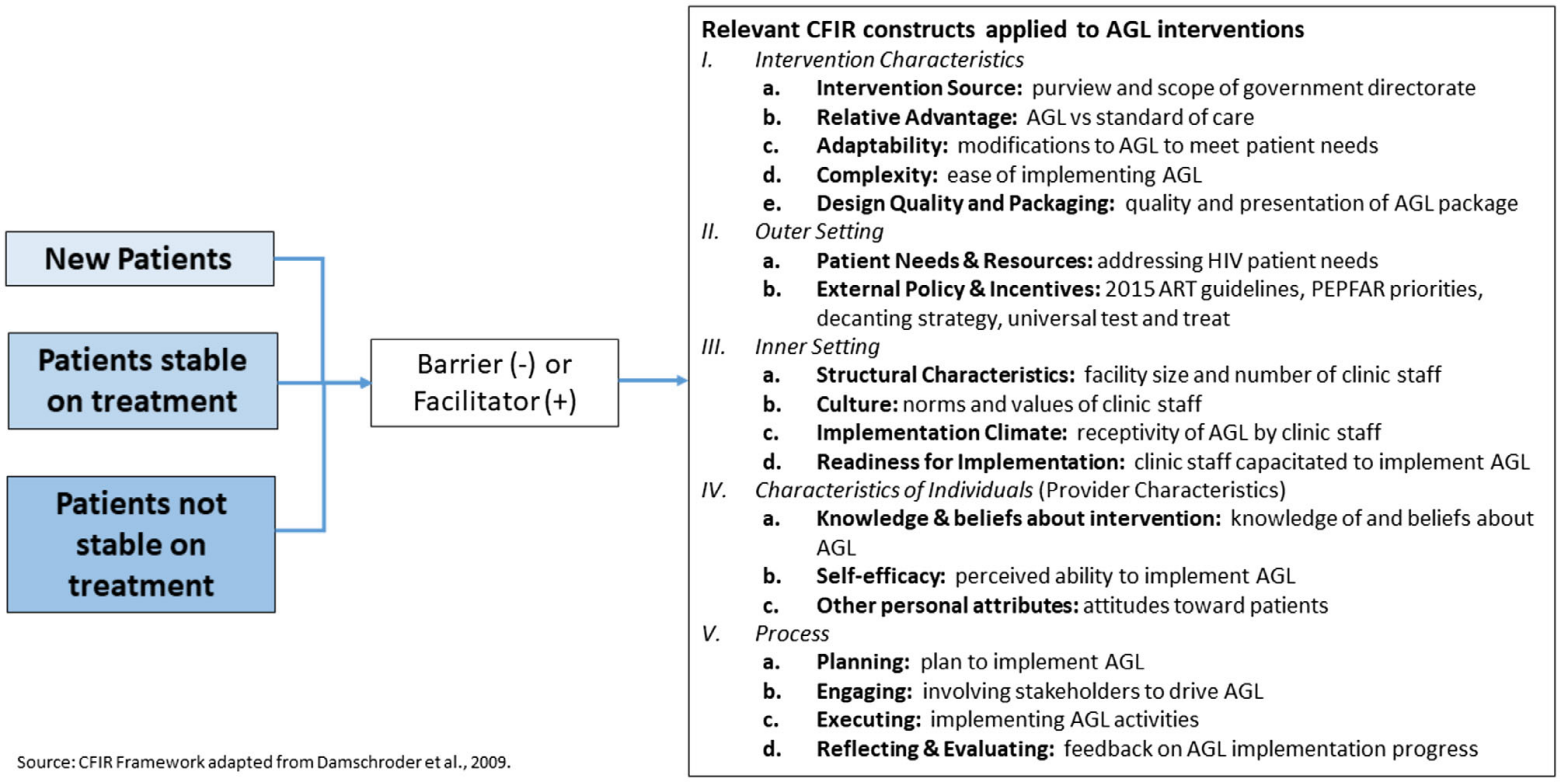
Application of the Consolidated Framework for Implementation Research to evaluate the National Adherence Guidelines interventions for new patients, patients stable on treatment and those patients who are not stable on treatment.

Figure 1 illustrates how we linked responses about AGL interventions to constructs in the CFIR (Figure 1) in order to guide and organize our interpretation of results. For example if a patient not stable on treatment perceived that EAC had more benefits than previous counselling, it would be coded under EAC and then as positive under “Relative Advantage,” because the respondent regarded the intervention as better than the standard of care.

### 2.4 Study sample

FGDs were conducted among three groups of patients at each of the eight study sites: (1) recently initiated treatment; (2) stable on treatment 12 months after initiation (defined by two consecutive viral loads that were undetectable and (3) not stable on treatment ≥3 months after initiation (defined by an unsuppressed viral load >400 copies/mL) or who had missed a visit by more than five days). Patients receiving the interventions (intervention sites) or those eligible to receive them (control sites) were identified from the records from the larger evaluation and invited to enrol in FGDs. Decentralized medicine delivery (DMD) management fell under the purview of a different government directorate than the other AGL interventions and consequently randomization of this strategy could not be preserved. Some control sites implemented DMD and some intervention sites did not. Where possible, we attempted to select stable FGD participants unexposed to DMD at control sites by confirming exposure prior to enrolment. The number of FGDs was determined a priori to provide a sufficient sample to reach thematic saturation [28, 29].

We conducted IDIs with a purposive sample of six providers per site responsible for implementing AGL or standard of care (e.g. lay counsellors, nurses, pharmacists, clinic operations managers and non‐clinical staff such as community health workers, data capturers and administrative clerks) and always the Clinic/Operations Manager. Four of the key implementing partner respondents in each district were purposively selected to be interviewed. Implementing partner respondents included district Department of Health (DOH) managers, technical experts and managers from partner NGOs. The number of provider interviewers per site was determined a priori to provide a sufficient sample to reach thematic saturation [28, 29]. The number of partners was selected to ensure representation from key implementing organizations.

### 2.5 Data collection and instrumentation

Data were collected November 2016 to October 2017 to allow patients enrolled in AGL interventions sufficient time to experience the interventions and to ensure that patient interaction for this sub‐study did not influence outcomes in the larger effectiveness evaluation. FGD guides were designed to elicit patient perspectives on: (1) quality and acceptability of interventions; (2) barriers to and facilitators of patient treatment adherence; (3) satisfaction and appropriateness of adherence services and (4) ways to improve the interventions. Semi‐structured IDI guides captured domains of effectiveness, patient‐centred/acceptability, accessibility, efficiency and equity. FGDs lasted between 53 and 129 minutes and IDIs 30 to 60 minutes.

### 2.6 Data management and analysis

All FGDs and all but one IDI were audio recorded, recordings were transcribed verbatim into the original local language and then translated and transcribed into English. Transcripts were checked for mistakes to improve reliability, then imported into NVivo 11^©^ (Doncaster, Australia), coded line‐by‐line and analysed using a content analysis approach [30]. Data analysis and interpretation was guided by the CFIR framework and stratified by AGL intervention patient type (new, stable, not stable on treatment) (Figure 1). Codes were identified a priori and additional codes were included as they emerged. To minimize researcher bias and reflexivity, two researchers coded each transcript, inter‐coder agreement was assessed using a Kappa coefficient, and coding was refined until agreement reached good correlation (>0.5). Findings were triangulated and contextualized with findings from the larger evaluation [14, 20].

Demographic data are presented for all respondent types. FGD and IDI results are presented in role‐ordered matrices with supporting illustrative quotes, stratified by intervention and control and by patient type. Key themes were not different between locations, therefore data are not stratified by site.

### 2.7 Ethical considerations

Ethical approval for the study was granted from the Boston University Institutional Review Board (IRB), (protocol H‐34197), and the University of the Witwatersrand Human Research Ethics (Medical) Committee in Johannesburg (protocol M150652). Data collectors were trained in qualitative interviewing techniques, Good Clinical Practice, research ethics and study procedures. We obtained written informed consent from each respondent.

## 3 Results

### 3.1 Sample characteristics

We conducted 24 FGDs (N = 156). One FGD was conducted with each of the three patient types at each of the eight study sites. Two‐thirds of respondents were between 30 and 49 years old (Table 2). Intervention sites had more female (71% vs. 57%) and slightly more married (28% vs. 22%) participants. A total of 48 providers participated in IDIs, 24 from intervention sites and 24 from control sites. The majority were female (81%) and over half were working as a clinical care provider (Table 2). Half of the 16 implementing partners were DOH staff and half from NGOs. Implementers oversee multiple facilities and are not categorized by study arm.

**Table 2:** Characteristics of patient focus group discussion and provider and implementer in‐depth interview respondents from the evaluation of South Africa’s National Adherence Guidelines for Chronic Disease

Patients	Control (N = 74)		Intervention (N = 82)		Total (N = 156)	
Characteristic	n	(%)	n	(%)	n	(%)
Age (n = 156)						
18 to 29	14	18.9	16	19.5	30	19.2
30 to 39	23	31.1	29	35.4	52	33.3
40 to 49	25	33.8	27	32.9	52	33.3
50+	12	16.2	10	12.2	22	14.1
Sex (n = 156)						
Female	42	56.8	58	70.7	100	64.1
Male	32	43.2	24	29.3	56	35.9
Marital status (n = 154)	73		81			
Never married	50	68.5	49	60.5	99	64.3
Married	16	21.9	23	28.4	39	25.3
Widowed/Divorced/Separated	7	9.6	9	11.1	16	10.4
Respondents by patient type						
Newly initiated treatment	17	23.0	22	26.8	39	25.0
Stable on treatment	35	47.3	31	37.8	66	42.3
Not stable on treatment	23	31.1	29	35.4	52	33.3
Respondents by province						
North West	17	23.0	19	23.2	36	23.1
Gauteng	20	27.0	23	28.0	43	27.6
Limpopo	20	27.0	21	25.6	41	26.3
KwaZulu Natal	17	23.0	19	23.2	36	23.1

Providers		Control (N = 24)		Intervention (N = 24)			Total (N = 48)		
Characteristic		n	(%)	n		(%)	n	(%)	
Sex (n = 48)									
Female		17	70.8	22		91.7	39	81.3	
Male		7	29.2	2		8.3	9	18.8	
Current role (n = 48)									
Clinical provider	13		54.2	12	50.0		25		52.1
Pharmacy staff	3		12.5	3	12.5		6		12.5
Non‐clinical facility staff	6		25.0	7	29.2		13		27.1
Outreach	2		8.3	2	8.3		4		8.3
Median no. of years working with facility/organization (range)	2.8 (1.0, 19.0)			3.5 (1.0, 16.0)			3.0 (1.0, 19.0)		
Median no. of years working with in current capacity (range)	3.5 (1.0, 26.0)			4.5 (0.0, 21.0)			4.0 (0.0, 26)		

Implementers					Total (N = 16)	
Characteristics					n	(%)
Sex (n = 16)						
Female	–		–		11	(68.3)
Male	–		–		5	(31.3)
Current role^a^ (n = 16)						
DOH staff	–		–		8	(50.0)
Implementing partners	–		–		8	(50.0)
Median no. of years working with facility/organization (range)	–		–		4.0 (3.0, 7.0)	
Median no. of years working with in current capacity (range)	–		–		4.0 (2.0, 27.0)	

^a^ Roles included CCMDD managers, trainers, technical experts, mentors, managers and coordinators.

### 3.2 Implementation outcomes

To organize results, key emerging barriers and facilitators by respondent type are illustrated by quotes (Table 3). Key barriers to implementation identified by patients (FGDs), providers and implementers (IDIs) are organized by salient CFIR construct in Table 4 (New patients), Table 5 (Stable patients) and Table 6 (Patients not stable on treatment) in order to facilitate comparison between respondent types. Generally, and not surprisingly, the majority of patient responses fell into the “Process” CFIR domain, as their experience is most impacted by their personal interactions with the interventions. From the providers’ and implementers’ perspectives, however, key barriers to implementation fell primarily within the CFIR domains of “Intervention Characteristics” (i.e. relative advantage and complexity, “Inner Setting” (i.e. readiness for implementation and implementation climate), and to a lesser extent on “Process.” Both FGD and IDI respondents converged on patient needs, particularly for stable patients. Detailed results are summarized below, organized by patient type.

**Table 3:** Qualitative responses from a cross‐sectional sample of patients, providers and implementers on barriers and facilitators to the implementation of South Africa’s National Adherence Guidelines for Chronic Diseases for new patients, stable patients and patients not stable on treatment or not adhering to care

Barrier/facilitator	Quote
1. Perspectives on interventions for new patients	
Facilitator	a) “…the experience I had with the one that I consulted with is that she went all out to make sure that I was comfortable and I understood what was expected of me as a patient and for my life to change for the better and be right … the female nurse that was assisting me played a motherly role, a champion role to me, I will not forget her.” – New patient, intervention
Barrier	b) “… I’m not satisfied yet because I think [fast‐tracking is] one of the things that leads to defaulting because they don’t get time to digest whatever they got today from the clinic. They knew the bad news if I can call it, yes, so they, they don’t understand… We don’t get enough time with them to make them understand, yes.” – Professional nurse, control
2. Perspectives on interventions for patients stable on treatment	
Facilitators	a) “The clubs are also assisting, because we no longer experience long queues. This is encouraging, because if you take your treatment consistently, you would ultimately qualify to belong to a club and that’s what we all crave for.” – New patient, intervention
	b) “We are saying to the patient: patient, you qualify because you are a good patient, there are these three decanting models. We want you to choose the one that suites you. Some are working, she might say she wants the SFLA [Spaced and Fast Lane Appointment] so that ‘I can come in quickly, collect my medication and go’. Some will say ‘I’ve got time, I will still want to come and socialise and be in the AC’, then she will go into that. Some will say, ‘No, no, no. I no longer want to even come into your facility. Let me go and collect it outside’. So, to me, I think it makes the patients to be responsible for their own choices, which is what primary health care says. Self‐actualization and self‐realization, we want them to be responsible for their own. Because if they are then responsible, then it will be easy for them to adhere.” – District‐based Department of Health implementer
Barriers	c) “I was not asked anything. I went to the clinic. When I arrived as usual, they said to me they are taking me out and adding me to the club of which I am happy with it.” – Stable patient, intervention
	d) “First, training. Second, a champion for each… like a [DMD] champion, or adherence club manager. Someone who’s going to be liable for that programme. So, when you go to a facility you know who to talk to about each problem. But currently when you go to a facility everyone is saying, ‘I don’t know anything about that, it’s not my job, I don’t know anything about that.’ But, if there is a sense of ownership in the facility, we’ll improve the implementation of adherence guidelines.” – Implementing partner
3. Perspectives on interventions for patients not stable on treatment	
Facilitators	a) “We motivate the patients. We motivate them to say, you still have a chance. If you are sick, you still have to come. So we motivate them, and we counsel them. It does work.” – Professional nurse, control
	b) “In previous cases, we used to do superficial things. Our counsellors now, they have been trained enough. They know that they have to take their time with the client and it’s not in our place to judge them. Because in previous cases, you find like the sister is accusing the client or judging the client. So now at least we have time, we try to understand their problems.” – Professional nurse, intervention
	c) “Once [patients who were traced] come to the clinic they become our first priority. We start with taking bloods if there is a need to take bloods. Then we send them back to enhanced counselling, because obviously these people are not compliant.” – Professional nurse, intervention
Barriers	d) “The counsellors do not guide you. Instead of advising you on how you should live a healthy life, they would instead shout at you. You tell them that you are currently stressed up, you are facing this and that problem, they would tell you that you are telling lies, that’s your own excuse, because they don’t know the background you come from.” – Stable patient, control
	e) “We are having the challenge of tracing the clients. We will find that some of them moved out without coming to the facility and ask for the referral letter or transfer letter. Because most of the people who are on ART are being employed at the nearest farms, and the production there is seasonal.” – Professional nurse, control
	f) “I think it’s also dangerous for the people who are tracing, because they tell us that in other places, only maybe a group of men will come out, so it is scary for the care workers. People don’t want their status to be known out there, so if you trace them, others become angry.” – Facility manager, intervention
	g) “By having a scanty number of community care givers, sometimes we get in difficult where there are no‐go areas where they can’t reach the other areas. They haven’t got transport, they walk.” – Facility manager, intervention

#### 3.2.1 Perspectives on interventions for patients newly initiated on treatment

Fast Track Initiation and Counselling, which initiated patients on ART after their first counselling session usually during their second clinic visit, was the primary AGL intervention for new patients. The standard of care – the 2015 ART guidelines and Universal Test and Treat (UTT) circular identified priority patients to start ART as soon as possible and within two weeks (Table 1) [13, 17, 31]. Patients in both arms valued knowing their status, supported testing for everyone and found counselling helpful during ART initiation (Table 4). The process of counselling was a major barrier to patients in both arms. Patients valued and desired frequent and substantive counselling, particularly after ART initiation (Table 3; quote 1a), and cited minimal attention from providers and misinformation as barriers to initiation (Table 4). In control sites, patients felt providers were not well informed nor well trained in ART initiation. Intervention respondents reported sub‐optimal counselling and privacy concerns as barriers to initiation.

Providers were sometimes confused between FTIC and UTT, and felt they were insufficiently prepared in terms of training and resources for either intervention. Consistent with patient perspectives, providers perceived pressure from government to rapidly rollout UTT, resulting in insufficient time to adequately prepare patients for initiation (i.e. physically, psychologically or emotionally). Some providers in control sites resisted expediting initiation and would at times deliberately not fast‐track patients without completed laboratory tests or sufficient counselling (Table 3; quote 1b).

**Table 4:** Facilitators and barriers to ART initiation and adherence for new patients eligible for the Fast track Initiation Counselling intervention under the South African National Adherence Guidelines mapped to relevant Consolidated Framework for Implementation Research constructs

Intervention		Control	
Facilitators	Barriers	Facilitators	Barriers
**Intervention characteristics:** **Design quality** Importance of counselling in UTT climate [IDI] **Relative advantage** FTIC was identified as faster and started patients on ART earlier than before [IDI]	**Intervention characteristics:** **Complexity** Some providers conflated UTT with FTIC, focusing on same‐day ART initiation [IDI] **Intervention source** Confusion around intervention source especially in UTT context [IDI] **Relative advantage** Some believe starting patients earlier is not beneficial for treatment outcomes [IDI]	**Intervention characteristics:** **Design quality** HIV education after testing [FGD] Home visits for HIV education and testing [FGD] **Relative advantage** UTT makes ART initiation faster [IDI] Advantage of treating patients before they are sick [IDI]	**Intervention characteristics:** **Complexity** Complexity of UTT in some cases resulted in patients not being initiated or potential defaulters [IDI]
**Outer setting:** **Patient needs** Eager to be informed of HIV status due to knowing someone with HIV [FGD] Received encouragement from friends to get tested [FGD] Openness with partner about HIV status [FGD] Belief that everyone should not be afraid and get tested [FGD] Addresses patient challenges with transport, as fewer visits are required before starting [IDI]	**Outer setting:** **Patient needs** Misinformed about HIV testing – only need testing if ill [FGD] Fear of getting tested because of stigma and knowledge of status [FGD] Fear of initiation because of stigma and side effects [FGD] Perceive lack of counselling and neglect after initiation [FGD] **External policy** Belief that pressure of rolling out UTT may de‐emphasize counselling during FTIC [IDI]	**Outer setting:** **Patient needs** Perceive a need for the government to change policy regarding HIV testing – everyone should be tested [FGD] Privacy when getting tested [FGD] Reduced trips to the clinic minimizes stigma [IDI] **External policy** Due to UTT push, increased testing and initiation is vital to reach 90‐90‐90 [IDI]	**Outer setting:** **Patient needs** Perceive that patients are not ready to start ARVs [IDI] **External policy** Belief that pressure of rolling out UTT may de‐emphasize counselling during FTIC [IDI]
**Inner setting:** **Implementation climate** Received ART education [FGD] Treatment collection dates were set up for patients [FGD] Perceive that patients like”fast‐tracking” because it is important to know your status and start treatment [IDI]	**Inner setting:** **Implementation climate** Lack of ART education [FGD] Perceive the clinic to be overcrowded and have long wait times [FGD] Unfamiliarity with the intervention [IDI] **Readiness for implementation** Unavailable stationery for record keeping [IDI]	**Inner setting:** **Implementation climate** Received ART education [FGD] Perceive intervention as important [IDI] Fast‐tracking works hand in hand with DMD [IDI] **Readiness for implementation** Algorithms or guidelines are available to help with ART initiation, often provided by the district development partner [IDI]	**Inner setting:** **Implementation climate** Limited encouragement from providers to take treatment [FGD] Some direct resistance to UTT [IDI] No training for UTT [IDI]
**Characteristics of providers:** **Personal attributes** Perceive some providers to be knowledgeable and encouraging [FGD] Expressed excitement about the FTIC intervention [IDI]	**Characteristics of providers:** **Personal attributes** Perceive some providers to be unfriendly and judgmental [FGD] **Self‐efficacy** Low readiness and unawareness of the intervention [IDI]	**Characteristics of providers:** **Knowledge & beliefs** Believe that patients are counselled and then are ready to initiate treatment [IDI]	**Characteristics of providers:** **Knowledge & beliefs** Perceive that some providers as not well informed/not trained in initiation of treatment [FGD] Perceive that patients are not ready to start ARVs without more counselling or laboratories [IDI] **Self‐efficacy** Low readiness to implement UTT [IDI]
**Process:** **Execution** Received counselling and believe it is beneficial [FGD] Believe in benefits of FTIC and perceive fast turnaround from testing to treatment initiation [FGD] Received support from family members or spouse/partner [FGD] Some providers described FTIC as a streamlined process [IDI]	**Process:** **Execution** Perceive a lack of privacy during counselling [FGD] Perceive a lack of counselling after testing [FGD] Perceive neglect from providers after initiation [FGD] Influence of myths from community surrounding side effects of ARVs and misinformation about testing [FGD] **Planning and engagement** Unclear distinction between and insufficient training on FTIC and UTT [IDI]	**Process:** **Execution** Process of HIV testing explained to patients and they were encouraged to take treatment [FGD] Perceive counselling to be helpful for relieving stress and fears [FGD] See benefits of taking ARVs and find it convenient [FGD] High quality of the standard of care for ART initiation including existing algorithms guiding the process [IDI]	**Process:** **Execution** Perceive lack of counselling after HIV testing or have only received counselling once [FGD] Perceive receiving a lack of or inconsistent information regarding treatment [FGD] Poor execution of consultation around UTT, the new approach could lead to increased defaulters [IDI] Do not fast track new patients because of belief that baseline labs and counselling is important [IDI] Insufficient training on UTT [IDI]

FGD, focus group discussions with patients; IDI: in‐depth interviews with providers and implementers.

#### 3.2.2 Perspectives on interventions for patients who are stable on treatment

Repeat Prescription Collection Strategies (RPCS) including clinic‐ or community‐based adherence clubs (ACs), decentralized medication delivery (DMD), and spaced and fast lane appointment systems (SFLA) were the primary AGL interventions for patients stable on treatment (Table 1). In control sites, adherence interventions implemented for stable patients included ACs and DMD, and interventions detailed in the 2015 ART guidelines (support groups, SMS reminders, buddy system and community outreach) (Table 1) [13, 17]. Because DMD was implemented at some control sites, respondents in both arms reported similar perceptions, and were generally positive about DMD and ACs. (Table 5).

Patients and providers concurred that DMD and ACs reduced queues and waiting times and decongested facilities (Table 3; quote 2a). DMD was especially helpful for employed or time‐constrained patients. Patients in both study arms found DMD easy, though many felt it lacked sufficient adherence support and health monitoring. Most patients in the intervention groups liked the social aspect of ACs, but some found them inconvenient. Others articulated a perception of exclusivity because not all stable ART patients were eligible (e.g. those with high blood pressure or tuberculosis), or were offered the opportunity to participate in ACs. Opinions differed on the process of implementation, specifically whether patients were given a choice of refill strategy. Implementers report it is foundational to offer a choice between RPCSs (Table 3; quote 2b), however, patients in both study arms felt they were not given a choice, and could only opt in or out of the singular RPCS offered to them (Table 3; quote 2c).

Providers and implementers found DMD difficult to manage and reported that medications were not always available for pick‐up when they should have been. Both faced early challenges implementing DMD, citing that: it was not well introduced at AGL initiation trainings with limited provider training on implementation of DMD; and the rollout of a separate strategy (the Decanting Strategy) by a different Department of Health directorate, which promoted just the RPCS interventions, caused further confusion. In addition, there was no clear ownership of the rollout of these interventions, and poor communication between facilities and DMD pick‐up points meant that scripting issues sometimes occurred preventing timely delivery of medications. Providers also found it challenging to monitor DMD patients for adherence, and perceived that patients felt “chased” from the facility, raising concerns about attrition. Suggestions to improve RPCS interventions included additional training, identifying champions and improving ownership of implementation (Table 3; quote 2d).

**Table 5:** Facilitators and barriers to ART adherence for stable patients eligible for Repeat Prescription Collection Strategies under the South African National Adherence Guidelines mapped to relevant Consolidated Framework for Implementation Research constructs

Intervention		Control	
Facilitators	Barriers	Facilitators	Barriers
**Intervention characteristics:** **Relative advantage** Reduced queues and waiting time [FGD & IDI] Perceive service as fast, fewer trips to the facility and more convenient times for those who work [IDI] ACs easier to manage compared to DMD and pickup is quick [IDI] Speed and convenience of DMD pickups is helpful [IDI] **Adaptability** Implementers able to adapt ACs – number of and types of patients [IDI] Existing ACs being aligned to the AGL – used to be collection points previously [IDI]	**Intervention characteristics:** **Relative advantage** Not all stable patients are eligible for ACs due to comorbidities [FGD] DMD not convenient for patients who desire health checks or want >1 month of medication [FGD] Risk of defaulters or data gaps as the system is streamlined between DMD and clinics [IDI] **Design quality** Routine collection excludes adherence activities unless patient is sick [FGD] Some confusion surrounding context of the intervention [IDI] **Intervention source** Challenges with the authority of implementation – clinic, pharmacy, or DOH Care and Support [IDI] **Complexity** Difficulty establishing cohorts early on when creating ACs [IDI]	**Intervention characteristics:** **Relative advantage** Believe reduced queues are an advantage of DMD [FGD] Found SMS reminders for DMD collection helpful [FGD] Perceive that ACs save time and money [FGD] Perceive DMDs as fast and convenient [IDI] **Adaptability** Believe DMD is flexible, especially if clinic has a stock‐out [FGD]	**Intervention characteristics:** **Relative advantage** Potential loss of connection between patients and providers [FGD] **Design quality** Routine collection excludes adherence activities unless patient is sick [FGD]
**Outer setting:** **Patient needs** Helpful for those who work [FGD] Patient privacy protected [FGD] Addresses issues of stigma [IDI] Reduces burden of distance because of fewer clinic visits [IDI]	**Outer setting:** **Patient needs** Clubs not convenient because of distance and association with HIV‐positive people, i.e. stigma, despite accommodation for all chronic medication pickup at club visit [FGD and IDI] Some desire to still have regular health checks [FGD]	**Outer setting:** **Patient needs** DMD is helpful for those who work because the hours are accessible and lack of queues [FGD] Report of dismantling DMD in favour of clubs as patients want to come in groups [IDI]	**Outer setting:** No codes mapped to this CFIR domain
**Inner setting:** **Implementation climate** Aware of the intervention options [FGD] Those not in ACs/DMD can be motivated to qualify through adherence; felt empowered and encourage to adhere [FGD] Perceive patients welcoming the various RPCS interventions [IDI]	**Inner setting:** **Implementation climate** Felt interventions were NOT compatible with clinic‐based services for chronic diseases such as hypertension or diabetes [FGD] Some felt not well informed about the interventions [FGD] Implementers perceive staff shortages [FGD] Challenges with resistance and buy‐in to the new interventions from providers [IDI] Difficulty of implementing RPCS in the context of other programmes [IDI] Limited available space for AC meetings [IDI] Perception that DMD was designed to chase patients from facility [IDI]	**Inner setting:** **Implementation climate** Felt the intervention was compatible with chronic diseases for patients who have diabetes, for example and still need regular clinic visits [FGD] Reports of non‐adherence to Viral Load Protocol [IDI]	**Inner setting:** **Implementation climate** Some felt the interventions were NOT compatible with chronic disease management [FGD] Some felt not well informed about DMD availability (ambiguity at control sites) [FGD] Believe that waiting a year for eligibility is too long [FGD] Concerns for patient files getting lost [FGD] Clinics are overcrowded [FGD]
**Characteristics of providers:** No codes mapped to this CFIR domain	**Characteristics of providers:** **Personal attributes** Some felt punished by providers if missed their appointment [FGD] Perceive bad attitude among providers [FGD and IDI] Fear that patients will default if they are left to pick up at DMDs [IDI]	**Characteristics of providers:** No codes mapped to this CFIR domain	**Characteristics of providers:** **Personal attributes** Some felt punished by providers if missed their appointment [FGD] Felt little support from providers unless sick [FGD]
**Process:** **Engagement** Some patients given a choice between AC and DMD [FGD] **Execution** Some preferred ACs because DMD only provided one month of medication [FGD] DMD generally described as an easy process [FGD] Partnerships between development partners and DOH [IDI] ACs running smoothly [IDI] Tracing loss to follow‐up is easier for clinic based RPCS [IDI] **Reflection** Implementers perceive need for more training at all levels of the facility [IDI] Implementers perceive lack of accountability and ownership [IDI]	**Process:** **Engagement** Some patients not given a choice between AC or DMD [FGD] **Planning and engagement** Early implementation challenges with DMD [IDI] DMD was not well introduced to facilities or health care providers [IDI] Multiple directorates guiding care, treatment and pharmaceutical services [IDI] No ownership of DMD by the facility staff; perceived as led by pharmaceutical services [IDI] **Execution** Felt implementation was going slowly [FGD] Not consulted or counselled about medication change [FGD] Some have not seen a change since the implementation of the AGL [FGD] Late or no delivery of medication at DMDs [IDI] Patients lose trust because of stock‐outs at DMDs [IDI] Perceived increase in lost to follow up because of communication gaps between facility and DMD [IDI] Illegible prescriptions and errors [IDI] Challenges monitoring DMD patients’ health and medication pickup [IDI]	**Process:** **Execution** Generally described as an easy process [FGD]	**Process:** **Engagement** Some patients not given a choice between AC or DMD [FGD] **Execution** Notable challenges with early implementation of DMD, including shut‐down DMD points and general problems with collecting at pharmacies (illegible prescriptions and errors) [FGD and IDI]

FGD, focus group discussions with patients; IDI, in‐depth interviews with providers and implementers.

#### 3.2.3 Perspectives on interventions for patients who are not stable on treatment

The primary AGL interventions for patients not stable on treatment were enhanced adherence counselling (EAC) and early tracing of all missed appointments (TRIC). The 2015 ART guidelines outline increased counselling efforts, home visits, pill counts and an emphasis on social support as the standard of care (Table 1). Overall, patients who are not stable on treatment at intervention sites were aware of the AGL interventions. They believed these approaches could benefit adherence behaviours but perceived crowded clinics and poor staff attitude as barriers (Table 6). Some patients from intervention sites were more positive, suggesting that some providers were helpful and respectful, and listened to patients, and that patient experience at the clinic is largely dependent on which staff are seen. Providers agreed with patients that staff shortages and long queues at the clinic were challenges but also believed good patient–provider relationships facilitate adherence. Providers trained in EAC felt better equipped to counsel patients and, in both arms, enhanced counselling was seen as beneficial when delivered well (Table 3; quotes 3a and 3b).

Tracing emerged as a priority for providers but implementation was a major challenge in both study arms. Providers noted having inaccurate patient contact information (Table 3; quote 3e) and mentioned safety concerns when physically tracing patients; staff shortages; and an inability to access lists of patients requiring tracing (Table 3; quotes 3f, 3g). Control sites mentioned that even when tracing is successful, the clinic is full and patients cannot be seen promptly. Some intervention sites reported prioritizing traced patients (Table 3; quotes 3c).

**Table 6:** Facilitators and barriers to ART adherence for patients not stable on treatment or not adhering to care eligible for Enhanced Adherence Counselling or Early Tracing interventions under the South African National Adherence Guidelines mapped to relevant Consolidated Framework for Implementation Research constructs

Intervention		Control	
Facilitators	Barriers	Facilitators	Barriers
**Intervention characteristics:** **Relative advantage** Perceive EAC to be better than previous counselling [IDI] See advantage of tracing to get missed visit patients back [IDI] Believe adherence guidelines are more structured and comprehensive than previous SOPs [IDI]	**Intervention characteristics:** **Complexity** Perceive degree of difficulty in implementing the interventions [IDI] Safety concerns surrounding tracing – encounter angry patients, only men answer the door, no transport, homes that have aggressive dogs [IDI] **Design quality** No social media or phone outreach [IDI]	**Intervention characteristics:** **Design quality** Having a routine clinic visit is helpful [FGD] Believe individual counselling gives privacy, educates and reassures patients [IDI]	**Intervention characteristics:** **Complexity** Challenging to trace patients because of wrong addresses and perceive as dangerous for women (can be harassed or attacked) [IDI] Challenging to recruit patients for support groups [IDI] Costs – providers use money out of pocket [IDI] **Design quality** ART communication material is insufficient [FGD] No support groups available for unstable patients [FGD] No social media or phone outreach [IDI] No support groups [IDI]
**Outer setting:** **Patient needs** Perceive benefits of taking ARVs [FGD] Support groups helpful because providers give inadequate attention [FGD] Perceive benefits of assigned dates to collect medication [FGD] Perceive benefits of SMS reminders for appointments and medication collection [FGD] Believe support groups, EAC and home visits are addressing patient needs; implementers emphasize ensuring patients understands the benefits of ART [IDI] Believe counsellors are now better trained and can address patient concerns [IDI] **External policy** Facility managers more motivated because they know they are being monitored by the district [IDI]	**Outer setting:** **Patient needs** Perceive needs and concerns not met by clinic, given inadequate information [FGD] Not aware of SMS/phone reminder system, believe that it would be useful [FGD] Dislike support groups and prefer individual counselling for privacy, not ready to disclose status [FGD] Patients complain about and do not attend support groups because of length of stay and lack of food [IDI] Challenges with tracing because patients often move or are scared to come back to the clinic because of missed appointment [IDI] **Cosmopolitanism** Challenges with home visits, because many other NGOs do the same [IDI]	**Outer setting:** **Patient needs** Encouraged to take ARVs and write down appointment dates [FGD] Find support group beneficial and comforting, easier to collect medication [FGD and IDI] Feel providers are attentive during medication collection [FGD] Missed visit tracing works when clinics work with community committees [FGD] Perceive benefits of assigned dates to collect medication [FGD] See benefits of counselling [IDI] Aware of why patients default: no food to take with ARVs, status disclosure, cannot come to clinic because of work or have to look after children [IDI] **Peer pressure** Aware of other facilities conducting home visits, believe it could be helpful [IDI]	**Outer setting:** **Patient needs** No encouragement from clinics [FGD] Perceive group counselling as not helpful, prefer individual counselling [FGD] Feel that providers do not listen to their suggestions [FGD] Challenges of tracing because of migrant populations [IDI] Have support groups but are aware that patients do not attend for many reasons: work, look after children, lack of food [IDI]
**Inner setting:** **Readiness for implementation** Access to resources and materials, training [IDI] **Relative priority** Recognize and believe in the importance of EAC and tracing [IDI] Prioritize patients who have been traced and return to the clinic [IDI] **Goals and feedback** Implementers are open to suggestions; ensure interventions work with clinic workflows [IDI] **Culture** Providers work with each other to help the patient [IDI]	**Inner setting:** **Structural characteristics** Perceive clinics to be overcrowded and inefficient [FGD] Challenges with tracing and long wait times at clinic because of staff or resource shortage [IDI] **Relative priority** Perceive clinics prioritize money over patient care, feel neglected by providers [FGD] **Compatibility** Challenge with integrating intervention activities into workflow, especially EAC and tracing – need to communicate interventions to patients [IDI]	**Inner setting:** **Relative priority** Perceive that if providers care, clinic will be efficient and patients cared for [FGD] Recognize and believe in the importance of the interventions, especially individual counselling, tracing and support groups [IDI] **Available resources** Have resources and materials to execute adherence activities [IDI]	**Inner setting:** **Structural characteristics** Challenges with tracing and patients returning to the clinic form tracing, because the clinic itself is overcrowded, cannot handle volume of patients [FGD and IDI] **Relative priority** Perceive poor encouragement from providers and feel neglected since initiation into treatment [FGD]
**Characteristics of providers:** **Personal attributes** Perceive helpful providers to be friendly, respectful, and who listen to patients [FGD] **Beliefs** Interested in interventions and value patient relationship [IDI] Implementers strongly believe in patient education at initiation [IDI] **Self‐efficacy** Believe providers are better trained now and are confident in executing the interventions [IDI]	**Characteristics of providers:** **Personal attributes** Perceive provider bad attitude and not interested in interventions, providers often shout at patients [FGD and IDI] Negative experiences with home visit – feel disrespected [FGD] **Self‐efficacy** Counsellors feel they lack training to give patients medication [IDI] **Beliefs** Not confident in tracing because of challenges [IDI]	**Characteristics of providers:** **Personal attributes** Perceive some providers to be considerate and listen to patients [FGD] **Beliefs** Believe adherence activities have been successful and can motivate patients to adhere [IDI] See advantages of tracing [IDI]	**Characteristics of providers:** **Personal attributes** Perceive provider bad attitude, providers often shout at patients [FGD] Perceive providers do not work hard or care about patients [FGD] Perceive poor quality service [FGD] Perceive counsellors as judgmental [FGD] **Beliefs** Not confident in tracing because of challenges [IDI]
**Process:** **Execution** Received counselling and saw benefits [FGD] Received HIV and nutrition education [FGD] Interventions executed and patients have responded well [IDI] **Engagement** Designated people to trace patients [IDI] **Reflection** Perceive SMS/phone calls and appointment cards to be helpful reminders [FGD] Perceive high quality of service at clinic, given adequate information [FGD] Perceive home visits to be helpful when the clinic is too far to collect medication, also receive HIV education [FGD] Implementers believe the intervention is constantly evolving; challenges at first but improved when AGL was streamlined into implementation plans [IDI]	**Process:** **Execution** Heard of support groups but have not actually seen them [FGD] Perceive that providers do not explain test results to patients [FGD] Intervention activities not happening according to plan [IDI] **Reflection** Perceive inadequate counselling, counsellors provide no guidance [FGD] Some patients do not like tracing and give wrong addresses – fear of status disclosure [IDI]	**Process:** **Execution** Home‐based caregivers visit households to deliver medications and check‐in [FGD] Received HIV and nutrition education [FGD] Received assigned dates to collect medication [FGD] **Engagement** Designated people to trace, do home visits, call and remind patients [IDI] **Reflection** Perceive interventions to be helpful and informational [FGD] Perceive benefits to receiving counselling [FGD] Attend support groups and find them helpful, would like to create one if it does not currently exist [FGD] See patients respond well to adherence activities ‐–counselling, reminders, tracing and support groups [IDI]	**Process:** **Execution** Perceive clinic visit process is not explained clearly [FGD] Patients not understanding importance of taking medication [FGD] Adherence activities not happening according to plan [IDI] **Reflection** Perceive inconsistent system for reminders of clinic visits and outreach, some feel clinic may have lost contact information [FGD] Perceive inadequate counselling, only received counselling once [FGD] Mixed feelings on home visits, some believe it may be helpful, while some would feel embarrassed [FGD] Patients dislike tracing and give wrong addresses and phone numbers – fear of status disclosure [IDI]

FGD, focus group discussions with patients; IDI, in‐depth interviews with providers and implementers.

## 4 Discussion

South Africa, like other countries in the region, embarked on an effort to make DSD a standard component of the HIV treatment programme. Implementing these models at scale, though, is complicated [32]. We identified a number of positive aspects in the implementation of the AGL in South Africa, but also elicited key challenges. Repeat prescription collection strategies (AC and DMD) were generally perceived positively by all concerned. The opportunity to avoid long queues and quickly pick up medicines, and to benefit from the social interaction and additional counselling and education offered at ACs, were key benefits [33, 34, 35, 36]. Providers generally felt adequately equipped to deliver ACs, likely benefiting from the long‐term experience of Médecins sans Frontiers implementing ACs and the training materials they developed [18, 37, 38]. This positive perspective is consistent with the results of the larger impact evaluation, which showed that patients enrolled in RPCS maintained, and in some cases improved, adherence and retention outcomes [14, 39], and with other evaluations of ACs in South Africa and Kenya [18, 33, 40, 41, 42, 43, 44, 45].

If early implementation successes are to be sustained as the AGL are taken to scale, a number of challenges need to be addressed. Providers expressed confusion around policies with overlapping targets [35]. FTIC was understandably conflated with the concurrent rollout of UTT [46, 47] and the 2015 ART guidelines which also provide guidance for fast‐tracking priority patients [48] given that both require expediting and/or combining counselling sessions to enable same‐day or rapid initiation of ART providers found it hard to make the distinction between these strategies. This unintended consequence underscores the need for clear, consistent communication channels when scaling complex interventions. The RPCS strategies for stable patients were confused with the implementation of the “Decanting Strategy” which promoted DMD and ACs under a separate national agenda [49, 50]. The “Decanting Strategy” was a national directive to decongest clinics and decant stable patients out of facilities resulting in the rollout of these interventions at non‐study sites within the districts [35]. EAC and TRIC are also offered, to some degree, under existing guidelines but prioritize slightly different groups of patients to those identified under the AGL. There was consequently confusion over which strategy should be prioritized and a lack of clarity on how AGL interventions differ from other policies. Providers were uncertain which strategy to implement or how to monitor implementation quality.

The complexity of DSD necessitates extensive and adequate training and support. Providers and implementers frequently felt they were not properly trained or sufficiently equipped to implement effectively across all types of patients, particularly with respect to DMD. Additionally, private pharmacists are an instrumental component of DMD, yet have traditionally not been engaged with public sector care and thus do not understand the intricacies of the DMD intervention. Though patients and providers largely perceived DMD to be beneficial, implementation challenges were seen as a risk to patient adherence and retention if medication was not available as expected [35].

Three main tasks are critical to the national implementation of the AGL intervention: (1) Ensuring providers engaged with DMD receive standardized, comprehensive training and mentorship around cohort creation and scripting; (2) Addressing scripting and medicine supply issues and (3) Providing integrated data systems so all providers can monitor and track patients both at the facility and pick‐up‐point. Expanding the services offered at external care points (both ACs and DMD pick‐up points), such as the ability to draw monitoring bloods, could further improve patient satisfaction and retention [51].

The preferences of HIV patients, such as appointments for clinic visits and medication collection, are an important driver of intervention uptake. Because not all patients were aware they had a choice between RPCS interventions, it is important that providers understand the available RPCS options and facilitate patients to select one that best meets their treatment need. Clear plans should also be in place for patients who transition between interventions to prevent attrition.

Providers faced most logistical challenges implementing EAC and TRIC. Providers underutilized viral load results because they could not access test results or because tests had not been done and those implementing TRIC did not have the lists of patients to contact or the resources to trace patients. These issues might be addressed by integrating data and coordinating resources between implementing partners and facilities. These results are aligned with the results of the larger evaluation which showed little difference in terms of suppression and retention outcomes between the intervention and control sites probably because there was little difference in the interventions that were implemented at each site [15]. Effectively managing patients not stable on treatment is critical to the broader HIV control strategy, but at the early learning sites, providers and implementers did not have the necessary training, tools, resources or support to do so.

Patients in all groups wanted more frequent and better quality counselling [34]. Similarly, providers were reluctant to implement FTIC, which should reduce the number of pre‐initiation counselling sessions, suggesting they felt patients were not ready for treatment initiation or that they were not comfortable initiating patients without completing counselling sessions. This demonstrated a lack of understanding about the FTIC counselling sessions and the continued counselling after initiation highlights the need for comprehensive AGL training within the context of multiple policies, strategies and guidelines.

These findings must be considered within the context of study limitations. First, this sub‐study was only conducted in eight health facilities. Because we reached saturation within our sample and the results are consistent with findings from the larger impact evaluation, however, we believe that the facilities selected are representative of others in their districts [14, 15]. Second, the views of those who did not initiate treatment, did not return to care after tracing, or were not offered or did not choose an RPCS are not included, limiting our ability to determine how AGL implementation might be further adapted to access these individuals. Third, we did not stratify results by sex nor were we able to differentiate between responses from patients who were categorized as not stable because they were not virally suppressed and those categorized as not stable because they had missed visits. Finally, confusion amongst respondents with other policies and guidelines, the fact that existing ART guidelines already made provisions for fast track initiation, enhanced adherence counselling and tracing and the concurrent implementation of AGL interventions at some control sites made it difficult to isolate the specific AGL interventions in this analysis. This may explain the similar themes across intervention and control groups.

## 5 Conclusions

It is likely that some early implementation challenges will resolve with time and experience. Others, however, such as training, mentorship and site readiness will require more investment when taking the AGL to scale. Ensuring that providers are sufficiently trained and equipped to implement the interventions, that adherence and treatment strategies and policies are harmonized and aligned, and that patients are adequately informed about their treatment choices and understand the benefits of the AGL strategies will be critical elements for their continued implementation success at scale.

## Competing Interests

The authors declare no conflicts of interest.

## Authors’ Contributions

NS, SP, MF and NFH conceptualized the study. NS and SP oversaw data analysis, prepared the draft manuscript and managed subsequent revisions. SP, JM and AH oversaw implementation of the study and all data collection and JM and RMF analysed the in‐depth interviews and focus group discussions, and contributed equally to writing the manuscript. AH, AM, SR, MF and NFH provided substantive feedback on several iterations of the manuscript. MP and YP oversaw implementation of the Adherence Guideline interventions and along with DW and MG provided feedback on the manuscript.

## Supporting information


**Table S1.** District profile for the four districts included in the evaluation of the South Africa’s National Adherence Guidelines for Chronic Diseases
