# Supplementary material for: “Patients are not the same, so we cannot treat them the same” – A qualitative content analysis of provider, patient and implementer perspectives on differentiated service delivery models for HIV treatment in South Africa
Source: J Int AIDS Soc. 2020 Jun 25;23(6):e25544. doi: 10.1002/jia2.25544 (PMC7316408; doi:10.1002/jia2.25544)
Supplement: Supplementary file 1 — Table S1. District profile for the four districts included in the evaluation of the South Africa’s National Adherence Guidelines for Chronic Diseases [file JIA2-23-e25544-s001.docx]

**Supplemental Table 1: District profile for the four districts included in the evaluation of the South Africa’s National Adherence Guidelines for Chronic Diseases**

| **Province** | **District** | **Setting** | **Un- employment 2016/17 (%)*** | **HIV testing coverage 2016/17 (%)*** | **Adult Viral load completion rate at 12 months -2015 (%)*^§^** | **Adult Viral load suppression rate at 12 months -2015 (%)*** |
| --- | --- | --- | --- | --- | --- | --- |
| Gauteng | Ekurhuleni Metropolitan Municipality | Urban | 28.8 | 33.2 | 84.5 | 75.5 |
| KwaZulu Natal | King Cetshwayo | Mixed rural | 34.7 | 44.0 | 80.4 | 92.9 |
| Limpopo | Mopani | Rural | 39.4 | 55.6 | 86.1 | 83.5 |
| North West | Bojanala Platinum | Mixed-rural | 30.7 | 31.1 | 75.1 | 83.4 |
| **Data source:* Massyn N, Padarath A, Peer N DC. *District Health Barometer 2016/17*; 2017. http://www.hst.org.za. Accessed July 9, 2018.  **^§^** Viral load completion rate is the proportion of patients who should have a viral load who have a viral load done. | | | | | | |
